# The Book World of Medicine and Science

**Published:** 1897-10-23

**Authors:** 


### The Book World of Medicine and Science.

The Röntgen Rays in Medical Work. By David Walsh, M.D.Edin., with an Introductory Lecture upon Electrical Apparatus and Methods, by J. E. Greenhill. (London: Baillière, Tindall, and Cox. 1897. Price 6s. nett.)

The steady progress made by this method of diagnosis makes it very desirable that the present state of our knowledge on the subject should be placed at the disposal of the profession in a handy and convenient form, and this is very well done in the volume before us. It is true that it is possible to employ the Röntgen rays for diagnostic purposes with very little knowledge of either electricity or photography, on the "touch the button and we do the rest" principle, but even one who deals with the subject in this way cannot well interpret his results unless he studies what has been done by others, and one of the advantages offered by this book lies in the admirable illustrations which are freely interspersed among its pages. The first part dealing with apparatus and methods is enough, although not much more than enough, to give a general idea how the rays are to be produced, how the skiagrams are to be taken, and how the fluorescent screen is to be managed. All the steps to be taken are very clearly explained. The second part, which is by Dr. Walsh, consists of an interesting demonstration, if we may so call it, of what actually can be done by the use of the apparatus. Much useful information is given in regard to many of the conditions illustrated. The various considerations which have to be borne in mind in localising foreign bodies are well described, and the necessity of bearing them in mind is very clearly shown. Altogether we think that the book will be found of much service by those for whom it is written. It will also be found interesting by many who have no intention of practising skiagraphy, for it is becoming necessary constantly to bear in mind the possibility of obtaining diagnostic help from the Röntgen rays, and every medical man should understand what they may be expected to do for him in this direction.

Hygienic Guide to Rome. By Dr. Mendini. Translated by John J. Eyre, M.R.C.P. (Scientific Press. Pp. 188. Price 2s. 6d.)

The number of English and American visitors to Rome is an ever-increasing one, and it would be much greater than it is were it not for the widespread belief that the capital of Italy is an unhealthy city. Story, the sculptor, combated this idea long ago, and after a protracted residence in the old city of the Cæsars assured us that the prejudice has not much, if any, foundation; but not being a medical man his words did not carry the weight they should have done. Now we are pleased to welcome Dr. Eyre's translation of Mendini's "Hygienic Guide," a perusal of which can hardly fail to satisfy everyone that Rome is not only the healthiest of all the large towns of Italy, but that it is one of the healthiest cities in Europe. No doubt much of the old prejudice arose from the fact that the country around Rome is not healthy, and when once an idea has become widespread it is proverbially difficult to eradicate it. Comparing the death-rates per thousand for the four years ending 1894 of the five largest towns in Italy, it is seen that Rome has the lowest with 21·4. Turin follows close with 21·5, Florence is third with 24·5. Milan is 25·4, and Naples has the unenviable notoriety of being the highest with 28·7. Further, comparing Rome with five European capitals during the decennium 1884-93, we find that London was lowest with a death-rate of 17·8 per thousand; Berlin second with 18·2; Rome third with 19·4; Vienna fourth with 22·8; and St. Petersburg highest with 31·4. The climate of Rome is admittedly most salubrious, even by those who fear the mists of the Campagna. The average number of wet days is only 96 in the year, and the mean temperature of January and December is 43·8 and 46 Fahrenheit respectively, while the mean minimum temperature of the same months is 39·1 and 40. July is the hottest month, with a mean temperature of 76. One chapter is devoted entirely to the water supply of Rome, and analyses of the various springs are given showing the purity of the supply. The sewage system of Rome is carefully described, and there are chapters on foods and on hospitals and charitable institutions. A chapter on "Rome as a Health Resort" is added by the translator, and it is one of the most interesting and instructive in the book. Altogether this hygienic guide may be confidently recommended to those who contemplate a sojourn in Rome, as it contains a large amount of useful information in a very accessible form not hitherto easily obtainable by the general reader.

The Insane in Private Dwellings and Licensed Houses. By J. F. Sutherland, M.D., F.R.S.E. (Edinburgh: E. and S. Livingstone. Glasgow: N. Adshead and Son. Price 1s.)

Without committing ourselves to the acceptance of the author's views in their entirety we think that this *brochure* might with advantage be read by all those who have to face the serious and almost appalling problem of providing accommodation for the pauper insane. The little book deals with the boarding out of the harmless insane in private houses. This has been carried out to such an extent in Scotland as to be known as the Scottish system. Considering the number of years which it has been in operation, it speaks volumes for the care with which the cases have been selected and the supervision under which they have lived that only one serious accident is reported. At the same time it is hardly fair to contrast this immunity with the records of an asylum. The two things are not comparable. In Great Britain (in Ireland it is almost non-existent) it is interesting to notice that this system finds most favour in the Celtic counties. In Scotland generally the high proportion of 23 per cent. is thus dealt with, but in the Highland counties this percentage rises to 38; Wales boards out 19 per cent., while England only provides for 6 per cent. in the same manner. It is also interesting to note what a high proportion of insane per 100,000 of the population is found in the Celtic counties of Scotland, and more especially in Argyll, as contrasted with the Lowland counties. When this is considered along with the class of cases boarded out, the student of racial characteristics might be tempted to ask whether he has here found evidence of degeneration in the Celt.

The conditions of life in the two countries are hardly the same, nor in England are they so favourable, but still we think that this method of dealing with harmless imbeciles and dements might be extended in the English counties. Under the Lunacy Act, 1890, such cases can be sent out to the care of friends and paid for by the asylum authorities, but they are still looked upon as being patients of the asylum. This being so, and remembering how sudden and serious may be the changes in a lunatic's mental condition, a superintendent is naturally averse to incur responsibilities when he is not in a position to exercise supervision over the cases. Patients are, however, frequently discharged altogether to the care of friends, who take charge of them without any monetary assistance from the asylum. Any attempt on a large scale to apply the Scottish system in England would probably meet with strenuous local opposition. The plan of granting allowances to recovering or recoverable patients who are sent out on trial might in some instances be taken advantage of more frequently.

It is undoubtedly held by many authorities that the building of large institutions, such as the London County Council has in hand, is a great mistake. They may, perhaps, be capable of more economical management than smaller institutions, but whether the patients gain corresponding benefits is a doubtful matter. No asylum should have more than 800 beds; indeed, we do not know whether it would not be wiser to reduce that number by 300.

